# Soluble urokinase plasminogen activator receptor predicts mortality in exacerbated COPD

**DOI:** 10.1186/s12931-018-0803-2

**Published:** 2018-05-21

**Authors:** Nina S. Godtfredsen, Ditte V. Jørgensen, Kristoffer Marsaa, Charlotte S. Ulrik, Ove Andersen, Jesper Eugen-Olsen, Line J. H. Rasmussen

**Affiliations:** 1 Department of Pulmonary Medicine, Amager and Hvidovre University Hospital, Hvidovre, Denmark; 2 Institute of Clinical Medicine, University of Copenhagen, Copenhagen, Denmark; 3 Palliative Unit, Gentofte and Herlev University Hospital, Herlev, Denmark; 4 Clinical Research Center, Amager and Hvidovre University Hospital, Hvidovre, Denmark

**Keywords:** COPD exacerbation, Mortality, Biomarker, Hospitalisation

## Abstract

**Background:**

The inflammatory biomarker soluble urokinase plasminogen activator receptor (suPAR) is elevated in severe acute and chronic medical conditions and has been associated with short-term mortality. The role of suPAR in predicting risk of death following an acute exacerbation of chronic obstructive pulmonary disease (AECOPD) has never been studied. We hypothesized that increased suPAR is an independent predictor of short-term mortality in patients admitted to hospital with COPD or acute respiratory failure.

**Methods:**

This retrospective cohort study from a university hospital in the Capital Region of Denmark included 2838 acutely admitted medical patients with COPD as primary (AECOPD) or secondary diagnosis, who had plasma suPAR measured at the time of admission between November 18th, 2013 to September 30th, 2015 and followed until December 31st, 2015. Primary outcomes were 30- and 90-days all-cause mortality. Association of suPAR and mortality was investigated by Cox regression analyses adjusted for age, sex, CRP values and Charlson comorbidity index.

**Results:**

For patients with AECOPD or underlying COPD, median suPAR levels were significantly higher among patients who died within 30 days compared with those who survived (5.7 ng/ml (IQR 3.8–8.1) vs. 3.6 ng/ml (2.7–5.1), *P* < 0.0001). Increasing suPAR levels independently predicted 30-day mortality in patients with COPD with a hazard ratio of 2.0 (95% CI 1.7–2.4) but not respiratory failure.

**Conclusions:**

In this large group of acutely admitted patients with COPD, elevated suPAR levels were associated with increased risk of mortality. The study supports the value of suPAR as a marker of poor prognosis.

**Electronic supplementary material:**

The online version of this article (10.1186/s12931-018-0803-2) contains supplementary material, which is available to authorized users.

## Background

Chronic obstructive pulmonary disease (COPD) is worldwide a leading cause of morbidity and mortality, posing a considerable socioeconomic burden to society [1]. COPD is a highly complex disease with symptoms not only related to the respiratory system. In many patients with COPD, the course of the disease will be progressive with a gradual decline in health status and increasing frequency and severity of acute exacerbations (AECOPD). This progressive course of the disease is associated with a substantial increase in all-cause mortality. In the clinical assessment of the severity of the disease, the GOLD classification is often applied [1]. However, improved risk prediction in patients with COPD would be of great value, in particular if it additionally would facilitate identification of terminal ill patients in need of enhanced preventive efforts, palliative care or other interventions.

Several biomarkers have, in studies of stable COPD, been associated with disease severity and mortality risk [2]. A history of frequent exacerbations of COPD is per se a marker of poor prognosis [3]. However, diagnostic and/or prognostic markers in unstable disease have so far provided variable results. In the ECLIPSE cohort, previous exacerbations, older age, high white blood cell count, emphysema, severe airflow limitation and poor health status were associated with increased risk of future hospitalizations [4]. A meta-analysis of routine blood-tests in AECOPD found that anaemia, hypoalbuminemia and elevated levels of the cardiac biomarkers pro-BNP and troponin-T, but not high-sensitivity C-reactive protein (hs-CRP) predicted mortality from COPD [5]. By contrast, a recent systematic review found CRP to be the only robust biomarker showing consistently elevated levels in AECOPD compared with control groups [6]. Recently, a readmission and mortality risk prediction score, comprising previous admissions, extended dyspnoea grade, age and heart failure but no biomarkers, has been proposed [7].

Previous studies have shown that the urokinase plasminogen activator receptor (uPAR) is involved in the pathogenesis (both small airway fibrosis and emphysema) of COPD through complex molecular pathways and gene expression patterns [8–10]. Soluble urokinase plasminogen activator receptor (suPAR) is the soluble form of the uPAR receptor and can be measured in blood/plasma. suPAR is considered an inflammatory biomarkerand is elevated in both acute and chronic illness. It is a non-specific risk marker with a diagnostic cut-off value of approximately 3 ng/ml [11]. However, little is known about suPAR in relation to disease status and mortality in COPD. suPAR has previously in a small study been found to be superior to CRP and fibrinogen in the prediction of AECOPD and in monitoring treatment of exacerbation [12]. Furthermore, suPAR levels are increased in stable COPD compared to healthy controls [13]. Finally, suPAR has been shown to predict a range of chronic conditions and mortality in the general population as well as being associated with risk of readmission and mortality in a large group of acutely admitted medical patients, with AECOPD constituting a subgroup of these [14, 15].

In this study, we aimed to investigate the value of suPAR levels in plasma upon hospital admission as a prognostic biomarker for short-term mortality in COPD patients based on a large, unselected population of acutely admitted medical patients.

## Methods

### Study design and population

This retrospective, registry-based cohort study was carried out in the Acute Medical Unit (AMU), Copenhagen University Hospital Hvidovre, Denmark, and included acute medical patients admitted from November 18th, 2013, until September 30th, 2015. The patients were followed in national registries for up to 90 days. The AMU receives unselected, adult internal medical patients within all specialties, except medical gastroenterology and patients requiring isolation due to infections or suspected acute myocardial infarction in need of PCI. Upon hospitalization, all patients routinely have a set of standard blood tests analysed, including blood cell count, electrolytes, analyses of kidney- and liver function, and CRP. Since November 18, 2013, suPAR has been included in this biomarker panel as a routine analysis. All patients, who were admitted to the AMU throughout the described period and had suPAR measured as part of the admission blood samples, were included in the present study.

For each patient, an index admission was defined as the first admission in which the patient had suPAR measured. Data on plasma suPAR levels and other blood tests were obtained from the electronic hospital database LABKA via the Department of Clinical Biochemistry. Hospital admission and discharge as well as information on diagnoses were obtained from the Danish National Patient Registry (NPR), [16] and for this study we included patients with the International Classification of Diseases, 10th edition (ICD-10) diagnoses for COPD (J440, J441, J449) or respiratory failure (J96X) at the index admission. Data on vital status was obtained from the Civil Registration System, in which all Danish citizens are registered with a unique personal identification number [17]. Follow-up was 30 or 90 days. For this study, three COPD populations of interest were defined based on the patients’ diagnoses at the time of the index admission:

1. Patients admitted with acute exacerbation of COPD as the primary diagnosis during the index admission (*AECOPD)*
2. Patients admitted with a diagnosis of COPD registered as the primary diagnosis before or during the index admission (*any COPD*)
3. Patients admitted with respiratory failure as the primary diagnosis during the index admission and with a registration of COPD as secondary or any other order of diagnosis during the index admission (*Respiratory failure*)

Thus, patients belonging to the groups *AECOPD* and *Respiratory failure* are also included in *any COPD*. However, no patient appeared more than once in the analyses, i.e. readmissions were not included in the study.

### Outcomes and covariates

The primary outcomes were death from any cause within 30 or 90 days after admission, respectively. Secondary outcome was analysis of the interaction between levels of suPAR and CRP.

Covariates included in the adjusted analyses were age, sex, Charlson comorbidity index (Charlson score), and CRP.

The Charlson score was calculated for each patient as a measure of the patients’ comorbid conditions using a SAS macro, [18] and using the updated weights defined by Quan et al. [19] Assessment of the Charlson score was calculated from diagnoses registered in the NPR up to 2 years prior to the index admission.

### Measurements

Plasma suPAR levels were measured using the suPARnostic AUTO Flex ELISA kit (ViroGates A/S, Birkerød, Denmark) as described in detail previously [15]. The suPARnostic® ELISA measures the full length suPAR molecule (D1D2D3) as well as the cleaved suPAR molecule (D2D3).CRP was measured using a COBAS 6000 analyser (Roche Diagnostics, Mannheim, Germany).

### Statistical analysis

Continuous variables are presented as median and interquartile range (IQR) and categorical variables are presented as numbers (n) and percentages (%).

We used both univariate and adjusted Cox regression analyses to estimate the effect of suPAR on time to death. Results are presented as hazard ratios (HRs) with 95% confidence intervals (CIs). The adjusted analyses were controlled for age, sex, Charlson score, and CRP. For the Cox regression analyses, suPAR was log2-transformed or stratified in quartiles.

Survival for suPAR- or CRP quartiles is presented in Kaplan-Meier plots for each of the three COPD groups.

Finally, we performed ROC curve analyses for 90-days mortality in the three groups using DeLongs test for two correlated ROC curves (with and without suPAR).

SAS Enterprise Guide 7.11 (SAS Institute) and R 3.2.3 (The R Foundation for Statistical Computing) were used for statistical analysis. R 3.2.3 was used to create the figures. A *P* < 0.05 was considered to be statistically significant.

## Results

SuPAR was measured in > 95% of all acutely admitted patients. During the inclusion period, a total of 717, 2573 and 265 patients with *AECOPD*, *any COPD* and *Respiratory failure due to COPD*, respectively, were admitted to the AMU and had suPAR measured. The median age was 72.8, 73.4 and 73.4 years, respectively, and median length of stay was 3.2, 2.2 and 5.9 days, respectively. In all three groups there were slightly more women than men who were admitted with COPD or respiratory failure (Table 1). In the group with *AECOPD*, 6.4% died within 30 days from the index admission, and 13.4% died within 90 days. For patients with *any COPD* or *Respiratory failure* the numbers were 9.1, 14.4 and 20.8% and 26.8%, respectively.

**Table 1:** Patient demographics and covariates at the index admission in patients with COPD as primary diagnosis, underlying COPD and respiratory failure due to COPD

	AECOPD	any COPD	Respiratory failure
*N* (%)	717 (20.2)	2573 (72.4)	265 (7.4)
Men	311 (43.4)	1142 (44.4)	109 (41.1)
Women	406 (56.6)	1431 (55.6)	156 (58.9)
Age (years)	72.8 (64.0–79.9)	73.4 (63.6–81.3)	73.4 (65.9–78.0)
Charlson score	1.0 (0–11)	1.0 (0–11)	1.0 (0–11)
Length of stay (days)	3.2 (0.8–7.8)	2.2 (0.7–6.8)	5.9 (1.8–12.0)
CRP (mg/L)	24.0 (6.0–75.0)	15.0 (4.0–65.0)	26.0 (7.0–86.0)
suPAR (ng/mL)	3.5 (2.6–5.0)	3.8 (2.8–5.4)	3.8 (2.8–5.3)

Values are presented as percentages or median (IQR)

### High suPAR and CRP are associated with increased risk of mortality

**Fig. 1:**
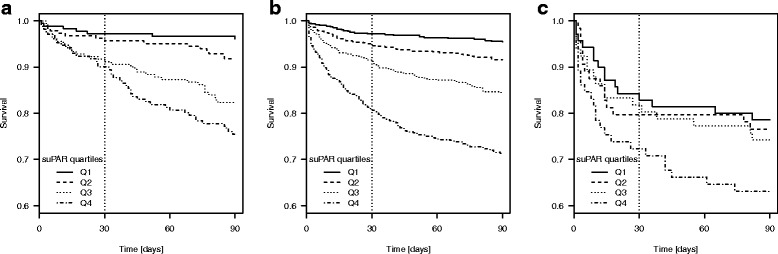
Kaplan-Meier plots of 90-day survival by suPAR quartiles. **a** Primary COPD, **b** Prevalent COPD, **c** Respiratory failure

**Fig. 2:**
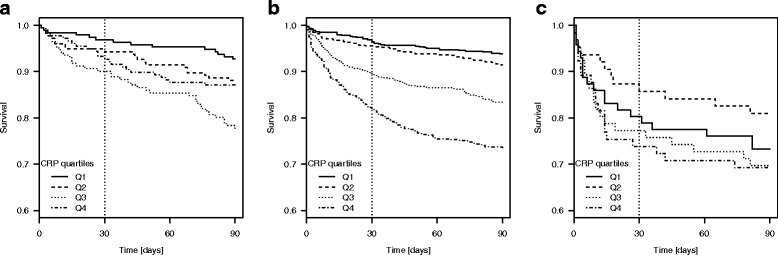
Kaplan-Meier plots of 90-day survival by CRP quartiles. **a** Primary COPD, **b** Prevalent COPD, **c** Respiratory failure

In all groups, except for patients admitted with *Respiratory failure* by 30 days of follow-up, median suPAR levels were significantly higher among those who died within 30 or 90 days of follow-up compared with patients who survived (Table 2). When stratifying the groups by suPAR quartiles, there was a significant relationship between increasing suPAR level and mortality by 30 or 90 days except in patients with *Respiratory failure* (Table 2, Fig. 1). Repeating the analyses by quartiles of CRP showed that increasing CRP was associated with both 30 and 90-day mortality in *AECOPD* only and with 90 days mortality in *any COPD* (Table 2, Fig. 2). For patients with acute respiratory failure and COPD there was, however, a trend towards increased 90-day mortality in the highest suPAR quartile/interval.

**Table 2:** Mortality rates in patients with AECOPD, any COPD, or Respiratory failure due to COPD, respectively, stratified according to suPAR and CRP quartiles^a^

	AECOPD			any COPD			Respiratory failure		
	Survived	Died	*p*-value	Survived	Died	*p*-value	Survived	Died	*p*-value
30-day mortality	671 (93.6%)	46 (6.4%)		2338 (90.1%)	235 (9.1%)		210 (79.3%)	55 (20.7%)	
suPAR (ng/ml), median (IQR)	3.5 (2.6–4.9)	4.5 (3.5–5.8)	0.0004	3.6 (2.7–5.1)	5.7 (3.8–8.1)	< 0.0001	3.8 (2.8–5.1)	4.1 (3.0–6.3)	0.14
CRP (mg/L), median (IQR)	21 (5–75)	46 (15–86)	0.03	13 (4–57)	71 (20–160)	< 0.0001	21.5 (6–80)	43 (8.5–115)	0.14
suPAR quartile 1	176 (97.2%)	5 (2.8%)		594 (97.2%)	17 (2.8%)		58 (82.9%)	12 (17.1%)	
suPAR quartile 2	176 (95.6%)	8 (4.4%)		634 (94.6%)	36 (5.4%)		51 (79.7%)	13 (20.3%)	
suPAR quartile 3	165 (91.2%)	16 (8.8%)		583 (91.2%)	56 (8.8%)		54 (81.8%)	12 (18.2%)	
suPAR quartile 4	154 (90.1%)	17 (9.9%)	0.01^b^	527 (80.7%)	126 (19.3%)	< 0.0001^b^	47 (72.3%)	18 (27.7%)	0.44^b^
CRP quartile 1	186 (96.9%)	6 (3.1%)		622 (96.4%)	23 (3.6%)		57 (80.3%)	14 (19.7%)	
CRP quartile 2	166 (94.3%)	10 (5.7%)		619 (95.5%)	29 (4.5%)		54 (85.7%)	9 (14.3%)	
CRP quartile 3	154 (90.1%)	17 (9.9%)		577 (89.6%)	67 (10.4%)		51 (77.3%)	15 (22.7%)	
CRP quartile 4	165 (92.7%)	13 (7.3%)	0.06^b^	520 (81.8%)	116 (18.2%)	< 0.0001^b^	48 (73.8%)	17 (26.2%)	0.40^b^
90-day mortality	621 (86.6%)	96 (13.4%)		2203 (85.6)	370 (14.4%)		194 (73.2%)	71 (26.8%)	
suPAR (ng/ml), median (IQR)	3.3 (2.6–4.6)	4.7 (3.7–6.2)	< 0.0001	3.6 (2.7–5.0)	5.4 (3.8–7.5)	< 0.0001	3.7 (2.8–5.0)	4.2 (3.0–6.3)	0.03
CRP (mg/L), median (IQR)	20 (5–75.5)	41 (13–73)	0.005	12 (3–54)	61 (16–130)	< 0.0001	19 (6–82)	43 (7–110)	0.11
suPAR quartile 1	174 (96.1%)	7 (3.9%)		583 (95.4%)	28 (4.6%)		55 (78.6%)	15 (21.4%)	
suPAR quartile 2	169 (91.9%)	15 (8.1%)		614 (91.6%)	56 (8.4%)		49 (76.6%)	15 (23.4%)	
suPAR quartile 3	149 (82.3%)	32 (17.7%)		540 (84.5%)	99 (15.5%)		49 (74.2%)	17 (25.8%)	
suPAR quartile 4	129 (75.4%)	42 (24.6%)	< 0.0001^b^	466 (71.4%)	187 (28.6%)	< 0.0001^b^	41 (63.1%)	24 (36.9%)	0.18^b^
CRP quartile 1	178 (92.7%)	14 (7.3%)		605 (93.8%)	40 (6.2%)		52 (73.2%)	19 (26.8%)	
CRP quartile 2	155 (88.1%)	21 (11.9%)		593 (91.5%)	55 (8.5%)		51 (81.0%)	12 (19.0%)	
CRP quartile 3	133 (77.8%)	38 (22.2%)		537 (83.4%)	107 (16.6%)		46 (69.7%)	20 (30.3%)	
CRP quartile 4	155 (87.1%)	23 (12.9%)	0.0004^b^	468 (73.6%)	168 (26.4%)	< 0.0001^b^	45 (69.2%)	20 (30.8%)	0.41^b^

^a^The cut-offs for the suPAR quartiles in each population were as follows: AECOPD: Q1: ≤2.6 ng/ml, Q2: 2.7–3.5 ng/ml, Q3: 3.6–5.0 ng/ml, Q4: > 5 ng/ml. Any COPD: Q1: ≤2.7 ng/ml, Q2: 2.8–3.7 ng/ml, Q3: 3.8–5.3 ng/ml, Q4: ≥5.4 ng/ml. Respiratory failure: Q1: ≤2.8 ng/ml, Q2: 2.9–3.8 ng/ml, Q3: 3.9–5.3 ng/ml, Q4: ≥5.4 ng/ml

The cut-offs for the CRP quartiles in each population were as follows: AECOPD: Q1: < 6 mg/l, Q2: 6–23 mg/l, Q3: 24–74 mg/l, Q4:≥75 mg/l. Any COPD: Q1: < 4 mg/l, Q2: 4–14 mg/l, Q3: 15–64 mg/l, Q4: ≥65 mg/l. Respiratory failure: Q1: < 7 mg/l, Q2: 7–25 mg/l, Q3: 26–85 mg/l, Q4: ≥86 mg/l.

^b^Chi^2^-tests by increasing suPAR and CRP-quartiles

### suPAR levels independently predict mortality risk

The survival analyses showed that suPAR is an independent predictor of 30- and 90-day mortality in patients admitted with*AECOPD or any other medical condition with underlying COPD*. In the multivariable models adjusted for age, sex, Charlson score and CRP, a doubling of the suPAR values (log2-transformed), gave HRs of 1.83 (95% CI: 1.21–2.79, *p* = 0.005), 2.17 (95% CI: 1.62–2.89, *p* < 0.0001) and 2.02 (95% CI: 1.69–2.41, *p* < 0.0001), 1.91 (95% CI: 1.66–2.20, *p* < 0.0001) for death by 30- and 90 days in the two groups, respectively (Table 3). Similarly, in the fully adjusted models by quartiles of suPAR, the HRs were higher in the highest suPAR quartiles compared with the lowest (Table 3). Age, sex and Charlson score were also independent risk factors for mortality in all three groups, whereas CRP only predicted subsequent mortality in the largest population (*any COPD*). When CRP was entered as a linear variable in the analyses, *p* < 0.0001 for 30- and 90-day mortality, and when CRP was divided by quartiles HRs in the highest quartiles compared to the lowest were 3.27 (95% CI: 2.06–5.20, *p* < 0.0001) and 2.90 (95% CI: 2.03–4.15, *p* < 0.0001) for 30- and 90-day mortality, respectively.

**Table 3:** Thirty- and 90-day mortality risk, expressed as hazard ratios, by suPAR levels, entered as either continuous (log-transformed) or in quartiles, at index admission in patients with AE COPD, any COPD and Respiratory failure due to COPD

Outcome	Analysis	Variable	AECOPD (*n* = 717)		any COPD (*n* = 2573)		Respiratory failure (*n* = 265)	
			HR (95% CI)	*p*-value	HR (95% CI)	*p*-value	HR (95% CI)	*p*-value
30-day mortality^b^	Univariate	Continuous	2.2 (1.5–3.2)	< 0.0001	2.5 (2.1–2.9)	< 0.0001	1.5 (1.0–2.1)	0.04
		1.quartile	1		1		1	
		2.quartile	1.6 (0.5–4.9)	0.42	2.0 (1.1–3.5)	0.02	1.2 (0.6–2.7)	0.62
		3.quartile	3.3 (1.2–9.0)	0.02	3.3 (1.9–5.6)	< 0.0001	1.1 (0.5–2.4)	0.82
		4.quartile	3.7 (1.4–10.1)	0.01	7.6 (4.6–12-6)	< 0.0001	1.8 (0.9–3.7)	0.12
	Multivariate^a^	Continuous	1.8 (1.2–2.8)	0.005	2.0 (1.7–2.4)	< 0.0001	1.3 (0.8–2.0)	0.23
		1.quartile	1		1		1	
		2.quartile	1.2 (0.4–3.8)	0.73	1.5 (0.8–2.6)	0.20	1.2 (0.5–2.7)	0.66
		3.quartile	2.3 (0.8–6.6)	0.11	1.8 (1.1–3.2)	0.03	0.8 (0.4–1.9)	0.65
		4.quartile	2.0 (0.7–5.7)	0.20	3.4 (2.0–5.9)	< 0.0001	1.3 (0.6–2.8)	0.50
90-day mortality^c^	Univariate	Continuous	2.5 (2.0–3.3)	< 0.0001	2.3 (2.1–2.6)	< 0.0001	1.5 (1.1–2.2)	0.01
		1.quartile	1		1		1	
		2.quartile	2.1 (0.9–5.3)	0.1	1.9 (1.2–2.9)	0.007	1.1 (0.6–2.4)	0.72
		3.quartile	4.9 (2.2–11-1)	0.0001	3.6 (2.4–5.4)	< 0.0001	1.0 (0.5–2.1)	0.98
		4.quartile	7.1 (3.2–15.8)	< 0.0001	7.3 (4.9–10.8)	< 0.0001	1.6 (0.8–3.1)	0.20
	Multivariate^a^	Continuous	2.2 (1.6–2.9)	< 0.0001	2.3 (2.1–2.6)	< 0.0001	1.4 (1.0–2.1)	0.06
		1.quartile	1		1		1	
		2.quartile	1.8 (0.7–4.5)	0.19	1.5 (0.8–2.6)	0.20	1.1 (0.6–2.4)	0.72
		3.quartile	3.9 (1.7–8.9)	0.001	1.8 (1.1–3.2)	0.03	1.0 (0.5–2.1)	0.98
		4.quartile	4.3 (1.9–9.9)	0.0005	3.4 (2.0–5.9)	< 0.0001	1.6 (0.8–3.1)	0.20

^a^Adjusted for age, sex, Charlson score and CRP

^b^Number of events were 45, 227 and 52 in AECOPD, any COPD and Respiratory failure, respectively

^c^Number of events were 93, 358 and 67 in AECOPD, any COPD and Respiratory failure, respectively

The ROC curve analyses confirmed that there were significant differences for the AUC, when suPAR was added to age, sex, Charlson score and CRP in *the two COPD* groups but not for *Respiratory failure* (not shown).Calculations of the sensitivity and specificity of log-transformed suPAR yielded values of 0.78 and 0.49, respectively, for 30- days mortality in the largest patient group (Additional file 1: Table S1).

### Interaction between CRP and suPAR

To assess whether the association between suPAR levels and mortality was mediated through a concomitant increase in CRP, we performed interaction analyses with CRP as a linear, continuous variable and suPAR as either a continuous variable or log-transformed in separate analyses for all three groups and for both 30- and 90-day mortality. There was no significant interaction between the two biomarkers except for 90-day mortality in *the largest group* when entering suPAR as a continuous variable (*p* = 0.04) (data not shown).

## Discussion

This retrospective cohort study in a population of patients admitted to a University hospital with AECOPD or any acute medical condition with underlying COPD showed that the inflammatory biomarker suPAR was independently associated with all-cause mortality. Furthermore, we showed that median suPAR levels were higher among those who died within 90 days of admission compared with those who survived in all three groups of respiratory patients. Interaction analyses showed that these effects were not mediated through concomitantly elevated CRP, although this biomarker was also independently associated with mortality, but less consistently compared to suPAR. This is consistent with previous findings and the value of CRP in combination with other biomarkers as indicators of poor prognosis in COPD has also recently been confirmed in a study comprising patients from the COPDGene- and the ECLIPSE cohorts [6, 20].

To our knowledge, this is the first large-scale study investigating the relationship between this recently introduced prognostic biomarker and COPD. The mortality rate in the present study did not differ from other studies of mortality in relation to severe AECOPD [21]. The magnitude of the association corresponds well with other studies that specifically have addressed the role of suPAR as prognostic indicator of mortality in acutely ill patients [15, 22, 23]. However, the vast majority of previous studies have investigated suPAR in cohorts of patients with systemic inflammatory response syndrome (SIRS) or sepsis in an intensive care unit setting, which makes comparisons of the present study with previous findings challenging. The sensitivity and specificity of suPAR regarding mortality in the present study is comparable with pooled values from a recent systematic review of suPAR and bacterial infections [24]. A very recent study of chronic heart failure (CHF), a condition which has the chronic, systemic inflammation in common with COPD, found that elevated suPAR levels approximately doubled the risk of subsequent mortality during 3 years of follow-up [25]. This was, however, stable patients in an outpatient setting. A Danish study of 449 patients consecutively admitted to hospital with chest pain and followed for up to 6.6 years found that suPAR was associated with increased all-cause mortality independent of other variables reflecting myocardial ischaemia (HR 1.93) [26]. Only one small cross-sectional study comprising 43 patients has measured suPAR upon hospitalisation with AECOPD and found it to be higher than in healthy controls [12] and also that suPAR correlated negatively with level of FEV_1_. Furthermore, suPAR levels decreased after 7 days, but whether this was a treatment effect or occurred spontaneously remains unknown. Two other small studies of stable COPD patients matched with healthy control subjects found opposite effects regarding suPAR levels [13, 27]. Thus, the value of adding suPAR to routine blood samples in hospitalised AECOPD for subsequent risk prediction has so far been largely unexplored. It has previously been demonstrated that the cell-bound form of suPAR (uPAR) is highly expressed in the small airway epithelia of patients with COPD compared with normal controls and that increased uPAR expression in the small airway epithelium of patients with COPD participates in an active epithelial-mesenchymal transition process [8, 9]. Assuming that increased uPAR expression results in increased uPAR cleavage and suPAR generation, the observations by Wang and co-workers are supported by our observation of increased suPAR and worse prognosis in COPD.

Given the pathophysiological mechanisms of most cases of AECOPD, namely a flare-up in acute local and systemic inflammatory mediators and airway bacterial or viral infection, [28] a high level of an unspecific inflammatory biomarker such as suPAR is expected. As suPAR is a very stable biomarker, it may be less affected by the exacerbation and more reflective of the underlying chronic condition, which may explain its high prognostic power in AECOPD.

In AECOPD no single biomarker or clinical test, apart from sustained respiratory acidosis measured in an arterial puncture, can accurately predict the immediate or short-term (less than 3 months) outcome [29]. Previous studies have proposed either a panel of biomarkers, a composite measure of clinical features and blood tests e.g. as a “prediction score tool” or simply an assessment of demographic variables for determining the risk for hospital readmission or death from COPD [2–7]. In terms of novel biomarkers of prognosis in COPD, the study by Stolz et al. [2] found that simultaneously elevated plasma levels of the three biomarkers adrenomedullin, arginine vasopressin and atrial natriuretic peptide were associated with increased mortality risk during a 5-year follow-up in patients with stable COPD. An assessment of adrenomedullin alone or in combination with composite measures such as age, grade of dyspnea and degree of airflow obstruction conveyed an increased mortality risk comparable to the observations in the present study [30]. However, the patients in this study were derived from several cohorts of patients who also had stable disease. Furthermore, a number of the scoring tools that have been developed for predicting future risk of readmission or death from AECOPD are quite complex or include measures of cardiovascular comorbidity, which may not be readily accessible in the acute setting [7, 31]. None of the scoring systems seem to agree upon, which few key parameters are considered superior in the prediction of severe and/or fatal incidents of AECOPD. The latest publication proposes an 8-item scoring tool for predicting 1-year mortality after a first hospitalization for AECOPD [32]. The variables associated with increased risk included older age, male sex, having a severe exacerbation, longer duration of the disease and hospital stay, prior hospital admissions, a diagnosis of cancer and a higher Charlson score. A number of these were already predefined as important confounders and included as covariates in the present study [15]. Interestingly, a study aimed at predicting 12-month mortality in elderly patients presenting to the emergency ward, by asking the attending physicians the so-called “surprise question” (“Would you be surprised whether this patient died in the next 12 months?”) suggested a fair sensitivity but poor specificity [33]. When combined with other determinants of unfavourable outcome, although not including any blood-based biomarkers, results remained unchanged.

For admission with respiratory failure and COPD as secondary diagnosis we found no statistical significant relationship between suPAR and mortality, although there was a tendency when entering suPAR as a continuous variable for death by 90 days (*p* = 0.06, Table 3). This may in part be due to the small numbers, especially when analysis is performed by suPAR quartiles. Nevertheless, this finding is somewhat unexpected and needs further investigation. Patients with acute, hypercapnic respiratory failure have an even higher risk of in-hospital or short-term mortality than patients belonging to either of the other groups, [29] which is why a stronger relationship would be anticipated in this group. The 30- and 90 days mortality rates were approximately doubled in the Respiratory failure group compared with the other groups, indicating that the negative results from the survival analyses represent a statistical lack of power rather than a true association.

Limitations of the present study include lack of data on smoking status as suPAR is confounded by smoking [34]. The Danish patient registries are validated and diagnoses are registered upon hospital discharge or in-hospital death but some inaccuracy may occur, although misclassification of the diagnoses in question is normally not a major source of bias [35]. Furthermore, this study did not discriminate the patients by other indicators of increased mortality risk such as number of previous exacerbations, need for non-invasive ventilation or referral to intensive care unit, level of FEV_1_, dyspnoea grade, BMI or socioeconomic status. However, regarding FEV_1_, the new GOLD guidelines recognize that a hospitalisation for COPD is more predictive of worsened prognosis than lung function [1]. Nevertheless we cannot rule out that patients with high levels of suPAR also had lower FEV_1_. Also, measurement of blood-eosinophils was only available in a minority of the patients. Another limitation is that suPAR values were only measured at hospital admission, and thus it was not possible to assess whether this biomarker changed according to therapy or disease course in general. In future studies of AECOPD it would also be useful to analyse how suPAR can contribute to already existing prediction scores for readmission and/or death.

## Conclusions

We have shown that suPAR levels are elevated in patients admitted to a large university hospital with COPD as primary or secondary diagnosis, who died within 30- or 90 days of follow-up compared with patients, who survived. The risk of death within 30- or 90 days after admission was doubled with increasing levels of suPAR for patients with AECOPD or any medical condition and underlying COPD, which is consistent with other studies. suPAR seems to be independent of- and better than CRP in discriminating an adverse outcome. It is therefore suggested that measurement of suPAR can be used as an indicator of poor prognosis in patients with more severe acute exacerbation of COPD.

## Additional file

Additional file 1: Supplementary table on the sensitivity and specificity of suPAR. Npv (negative predictive value), ppv (positive predictive value). (DOCX 14 kb)
