# Supplementary material for: Soluble urokinase plasminogen activator receptor predicts mortality in exacerbated COPD
Source: Respir Res. 2018 May 21;19:97. doi: 10.1186/s12931-018-0803-2 (PMC5963104; doi:10.1186/s12931-018-0803-2)
Supplement: Supplementary file 1 — Supplementary table on the sensitivity and specificity of suPAR. Npv (negative predictive value), ppv (positive predictive value). (DOCX 14 kb) [file 12931_2018_803_MOESM1_ESM.docx]

|  | sensitivity | npv | ppv | specificity |
| --- | --- | --- | --- | --- |
| AECOPD 30-day mortality | 0.777 | 0.969 | 0.094 | 0.486 |
| AECOPD 90-day mortality | 0.763 | 0.941 | 0.219 | 0.581 |
| Any COPD 30-day mortality | 0.629 | 0.950 | 0.177 | 0.710 |
| Any COPD 90-day mortality | 0.631 | 0.919 | 0.258 | 0.698 |
| Resp. failure 30-day mortality | 0.423 | 0.837 | 0.301 | 0.752 |
| Resp. failure 90-day mortality | 0.447 | 0.8 | 0.410 | 0.774 |

Supplementary table on the sensitivity and specificity of suPAR. Npv (negative predictive value), ppv (positive predictive value).
